# Mechanisms and treatment of obesity-related hypertension—Part 1: Mechanisms

**DOI:** 10.1093/ckj/sfad282

**Published:** 2023-11-13

**Authors:** Aneliya Parvanova, Elia Reseghetti, Manuela Abbate, Piero Ruggenenti

**Affiliations:** Department of Renal Medicine, Clinical Research Centre for Rare Diseases “Aldo e Cele Daccò”, Istituto di Ricerche Farmacologiche Mario Negri IRCCS, Bergamo, Italy; Unit of Nephrology and Dialysis, Azienda Socio-Sanitaria Territoriale Papa Giovanni XXIII, Bergamo, Italy; Research Group on Global Health, University of the Balearic Islands, Palma, Spain; Research Group on Global Health and Lifestyle, Health Research Institutte of the Balearic Islands (IdISBa), Palma, Spain; Department of Renal Medicine, Clinical Research Centre for Rare Diseases “Aldo e Cele Daccò”, Istituto di Ricerche Farmacologiche Mario Negri IRCCS, Bergamo, Italy; Unit of Nephrology and Dialysis, Azienda Socio-Sanitaria Territoriale Papa Giovanni XXIII, Bergamo, Italy

**Keywords:** hypertension, leptin, obesity, renin–angiotensin–aldosterone system, sympathetic nervous system

## Abstract

The prevalence of obesity has tripled over the past five decades. Obesity, especially visceral obesity, is closely related to hypertension, increasing the risk of primary (essential) hypertension by 65%–75%. Hypertension is a major risk factor for cardiovascular disease, the leading cause of death worldwide, and its prevalence is rapidly increasing following the pandemic rise in obesity. Although the causal relationship between obesity and high blood pressure (BP) is well established, the detailed mechanisms for such association are still under research. For more than 30 years sympathetic nervous system (SNS) and kidney sodium reabsorption activation, secondary to insulin resistance and compensatory hyperinsulinemia, have been considered as primary mediators of elevated BP in obesity. However, experimental and clinical data show that severe insulin resistance and hyperinsulinemia can occur in the absence of elevated BP, challenging the causal relationship between insulin resistance and hyperinsulinemia as the key factor linking obesity to hypertension. The purpose of Part 1 of this review is to summarize the available data on recently emerging mechanisms believed to contribute to obesity-related hypertension through increased sodium reabsorption and volume expansion, such as: physical compression of the kidney by perirenal/intrarenal fat and overactivation of the systemic/renal SNS and the renin–angiotensin–aldosterone system. The role of hyperleptinemia, impaired chemoreceptor and baroreceptor reflexes, and increased perivascular fat is also discussed. Specifically targeting these mechanisms may pave the way for a new therapeutic intervention in the treatment of obesity-related hypertension in the context of ‘precision medicine’ principles, which will be discussed in Part 2.

## Graphical Abstract

**Graphical Abstract:**
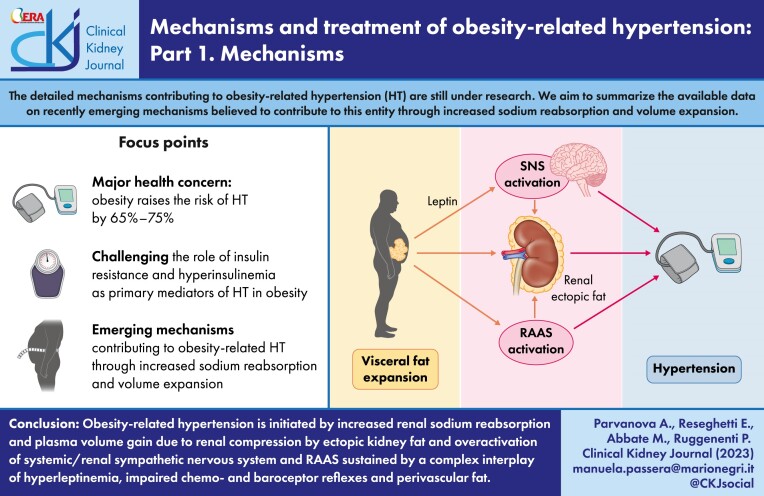

## INTRODUCTION

The prevalence of obesity, defined as a body mass index (BMI) ≥30 kg/m^2^, has nearly tripled over the past 50 years, reaching pandemic levels [1]. In 2016, WHO estimated that 39% of adults (1.9 billion) were overweight and 13% (over 650 million) of them were obese. Additionally, 340 million children and adolescents aged 5–19 years and 24 million children under the age of 5 years were also overweight or obese. Projections suggest that by 2030, 20% of the world's adult population (almost 1 billion persons) will be obese [2, 3]. The steady increase in obesity has led to a dramatic increase in its associated comorbidities, such as cardiovascular disease (CVD) [4], hypertension [5], type 2 diabetes mellitus (T2DM) [6], chronic kidney disease (CKD) [7], musculo-skeletal disorders [8] and several types of cancer [9], comorbidities that impose a major clinical and economic burden for healthcare providers worldwide.

Based on the relationship between higher blood pressure (BP) and increased risk of CVD [10], hypertension is currently defined as a BP of 130/80 mmHg or higher or self-reported antihypertensive medicine use [10]. The number of subjects with arterial hypertension has increased from 594 million in 1975 to 1.13 billion in 2015 [11], an increase that closely parallels the obesity epidemic. Elevated systolic BP is a major risk factor for CVD, the leading cause of death worldwide [12]. According to a systematic analysis by the Global Burden of Disease Study in 2019, high systolic BP accounted for 10.8 million deaths worldwide, making it the leading risk of death in females and second only to tobacco consumption in men in the Level 2 of the risk factors hierarchy, which includes 20 risks or clusters of risks [13]. Weight gain increases the risk of primary hypertension by 65%–75% [5], a correlation that is almost linear even at BMI <25 kg/m^2^ [14]. A gain of 1.7 kg/m^2^ BMI or 4.5 cm waist circumference is associated with a 1-mmHg increase in systolic BP [15, 16]. The association between body weight and hypertension first emerged in the Framingham Heart Study in the 1960s [17], however the mechanisms of this close relationship remained unknown until the second half of the 1980s when some epidemiological studies, inspired by the work of Vague [18] and coworkers observed that metabolic and cardiovascular complications were more frequently linked to obesity in subjects with ‘android’ obesity than in those with a ‘gynoid’ phenotype [19–21]. This observation highlighted the predominant pathogenic role of distribution of body fat (over its total amount) in the development of obesity-related hypertension which could explain the higher prevalence of hypertension in men despite the higher prevalence of obesity in women [22].

In 1988, Gerald Reaven hypothesized that insulin resistance was the key factor in a group of metabolic disorders, later called ‘Syndrome X’, which included impaired glucose tolerance (IGT), hyperinsulinemia, high triglycerides, low high-density lipoprotein cholesterol levels and hypertension [23, 24]. Then Norman Kaplan identified also central obesity as a key driver of CVD and defined the cluster of visceral obesity, IGT/insulin resistance, hypertriglyceridemia and hypertension as the ‘deadly quartet’ [25]. For more than 30 years, insulin resistance and hyperinsulinemia-dependent overstimulation of sympathetic nervous system (SNS) and increased renal sodium reabsorption have been considered as primary mediators of elevated BP in metabolic syndrome and obesity. However, strong experimental and clinical evidence is available to suggest that obese subjects can be normotensive despite severe insulin resistance and hyperinsulinemia [26]. Indeed, increased renal sodium reabsorption and consequent expanded plasma volume sustained by compression of the kidney by perirenal and intrarenal fat and systemic/renal SNS and renin–angiotensin–aldosterone system (RAAS) overactivation have been subsequently identified as major factors mediating the initiation of hypertension in obesity [5, 26]. On the other hand, it is clear that in the long term, insulin resistance and hyperinsulinemia, and consequent hyperglycemia and dyslipidemia, interact synergistically with elevated BP in the induction of renal and vascular injury that in a ‘vicious circle’ contribute to further worsening of hypertension and increased risk of renal and cardiovascular complications.

Part 1 of this review aims to summarize available evidence on new emerging mechanisms possibly involved in the pathogenesis of obesity-related hypertension, such as physical compression of the kidney by perirenal and intrarenal fat and overactivation of the SNS and RAAS in addition to the role of hyperleptinemia, impaired chemoreceptor and baroreceptor reflexes, and increased perivascular fat. Therapeutic interventions specifically aimed to target these mechanisms according to the ‘precision medicine’ principle, will be overviewed in Part 2 of the review.

## OBESITY, ADIPOSE TISSUE EXPANSION, GENETICS AND HYPERENSION

Obesity is a multifactorial disease that results from interactions between genetics and lifestyle [27, 28]. Heritability accounts for around 40% of cases of obesity [27, 29], while lifestyle factors account for the remaining 60% of cases, which allows obesity to be defined as a modifiable risk factor.

Body fat consists in large part of energy-storing white adipose tissue. However, there is also a component of metabolically active brown adipose tissue that usually does not exceed 3% of total body fat and is primarily involved in thermogenesis [30]. In adults, approximately 80% of white fat is stored subcutaneously and the remaining fat is located in visceral tissues. In obesity, body fat expands through hyperplasia of subcutaneous adipocytes and hypertrophy of visceral adipocytes, respectively. Paradoxically, the hyperplasia of subcutaneous adipocytes is partially protective against metabolic disorders even in severe obesity [31, 32]. However, when subcutaneous fat is maximally expanded, lipids start accumulating in visceral adipocytes, a process that is considered to be involved in inflammation, oxidative stress, lipotoxicity, insulin resistance, increased production of free fatty acids, cytokines [interleukin (IL)-1β, IL-6, tumour necrosis factor (TNF)-α], hormones [angiotensinogen, angiotensin II (AngII), leptin and resistin] and overall worsening of the atherogenic profile [33–36]. Therefore, visceral fat expansion is associated with increased cardiometabolic risk [31, 37] and waist circumference—taken as a marker of visceral adiposity—is currently considered as a more accurate indicator of obesity-related cardiometabolic risk than BMI, which is mainly a marker of subcutaneous fat accumulation [32, 38]. Furthermore, genome-wide association studies (GWAS) of BMI and waist-to-hip ratio found that loci for waist-to-hip ratio generally do not overlap with loci for BMI, suggesting independent regulations of fat distribution from total adiposity [39]. In addition to genetic factors, the expansion of subcutaneous adipose tissue is also influenced by diet, physical activity and sex, which may explain why the incidence and severity of obesity-related complications may differ among individuals with similar BMIs [40].

**Figure 1:**
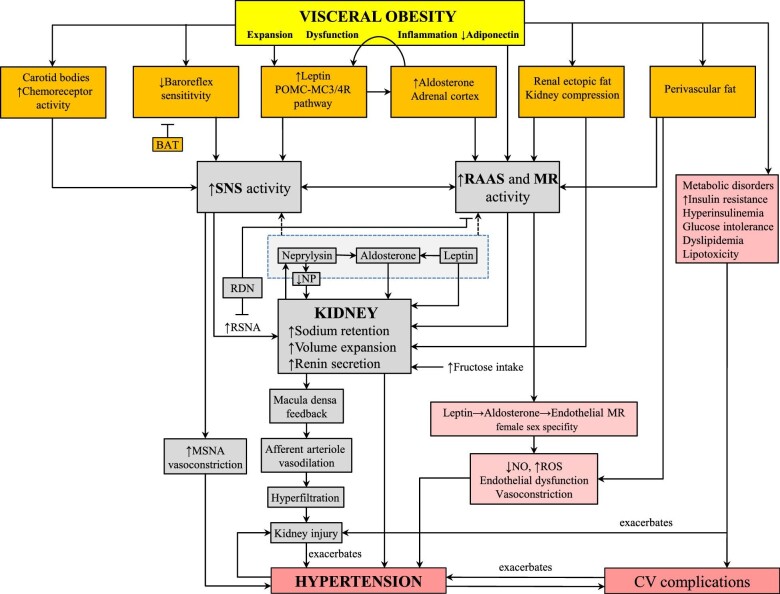
Putative mechanisms of obesity-related hypertension. BAT, baroreceptor activation therapy; CV, cardiovascular; MSNA, muscle sympathetic nerve activity; NP, natriuretic peptide; POMC-MC3/4R, pro-opiomelanocortin-melanocortin receptors; RDN, renal denervation; ROS, reactive oxygen species; RSNA, renal sympathetic nerve activity.

Obesity-related hypertension is sustained by a complex interaction between renal, neural, endocrine, vascular and other mechanisms (Fig. 1). In this interplay, multiple genes contribute to specialized functions that regulate BP, and many genes can possibly be involved in the development of obesity-related hypertension. GWAS have identified over 100 single nucleotide polymorphisms (SNPs) associated with BP phenotypes [41] and more than 300 SNPs associated with BMI, waist-to-hip ratio and other adiposity traits [39]. The mechanisms underlying the numerous epidemiological and genetic correlations among these obesity traits remain largely unknown. The largest association study of genomic inversions and comorbid disorders demonstrated the role of some polymorphic inversions as a major genetic contribution to the joint susceptibility to common diseases. In particular, a causal pathway was found in which obesity mediated the independent associations of 8p23.1 and 16p11.2 inversions with diabetes and hypertension [42]. A recent meta-analysis of 12 Mendelian randomization studies corroborated the high-level evidence for a causative effect of genetically predicted obesity on hypertension along 9 of 16 CVD-related outcomes. Notably, the association between obesity and hypertension was consistent between sexes [43].

### Diet, fructose and uric acid in obesity-related hypertension

Increased fructose consumption plays a role in the pathogenesis of obesity-related hypertension by inducing visceral fat expansion [44–52] and stimulating salt absorption in the small intestine and renal tubules (Fig. 1). The latter is achieved by the activation of the main carbohydrate-stimulated salt absorption molecules in the small intestine such as Na^+^/H^+^ exchanger 3 (NHE3), putative anion transporter 1 (PAT1) and sodium-glucose cotransporter (SGLT), and in the renal tubules such as NHE3, sodium chloride co-transporter (NCC), SGLT2 and epithelial sodium channel (ENaC) [53–55]. Furthermore, high fructose consumption and/or obesity increase uric acid production, which appears to contribute to hypertension through endothelial dysfunction [56, 57]. A recent review of uric acid in human disease pointed to the role of uric acid location [58]. While extracellular hyperuricemia is mainly associated with gout, nephrolithiasis and vascular calcification, experimental evidence strongly suggests that intracellular urate elevation is a key factor in the pathogenesis of primary hypertension by stimulating nicotinamide adenine dinucleotide phosphate (NADPH) oxidase, which increases oxidative stress in vascular smooth muscle and kidneys, contributing to the initiation and progression of hypertension [58, 59].

## RENAL ECTOPIC FAT, KIDNEY COMPRESSION AND PERIVASCULAR FAT

**Figure 2:**
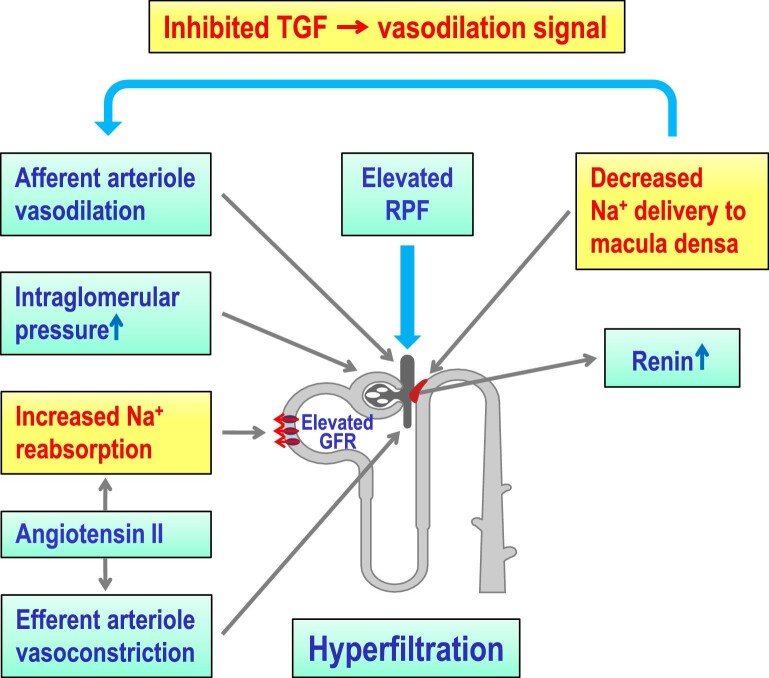
Mechanisms of hyperfiltration in obesity. TGF, tubule-glomerular feedback; RPF, renal plasma flow; GFR, glomerular filtration rate, Na^+^, sodium.

An important component of visceral fat is the renal ectopic fat, including the perirenal adipose tissue located between the renal capsule and renal fascia, and the renal sinus fat,encompassing the fat located at the medial border of the kidney closely associated with calyces, renal vessels, nerve fibres and lymphatic channels [60–64]. Excessive perirenal/sinus fat compresses the kidney, increases intrarenal pressure and reduces flow rate in the vasa recta and the loop of Henle (Fig. 1). These changes increase sodium reabsorption in the proximal tubule, decrease sodium delivery to the macula densa and inhibit the tubule–glomerular feedback causing preglomerular vasodilation, increased renal blood flow, glomerular hyperfiltration and renin secretion (Fig. 2). Moreover, perirenal/sinus fat produces adipocytokines such as leptin, resistin, IL-1β, IL-6, TNF-α, etc., which may also contribute to obesity-related hypertension by stimulating inflammatory processes and by activating the SNS and the RAAS [65–68]. Conversely, bilateral ablation or denervation of perirenal adipocytes produced a long-lasting decrease in BP in spontaneously hypertensive rats without effects in normotensive control rats [69]. Afferent nerves in perirenal fat were identified as a prohypertensive node that increases BP by suppressing calcitonin gene-related peptide (CGRP), which is a key endogenous BP-lowering mediator. Thus, modulation of perirenal fat afferent nerve activity may be a potential target for patients with reduced plasma CGRP or resistant hypertension [69]. Perirenal fat thickness, assessed by ultrasound evaluation, was higher in hypertensive than in normotensive patients and independently predicted systolic BP [70]. In obese hypertensive patients who underwent sleeve gastrectomy, there was a positive correlation between pre-intervention perirenal fat thickness and post-intervention reduction in antihypertensive drugs. Renal sinus fat, assessed by magnetic resonance imaging (MRI), was larger in obese and hypertensive subjects compared with lean and normotensive subjects, respectively [71]. Following bariatric surgery, a greater reduction in renal sinus fat was associated with a more effective amelioration of hypertension. Furthermore, the reported positive correlation between perirenal fat and creatinine [70] and the negative correlation between renal sinus fat and glomerular filtration rate estimated by the serum creatinine–based Chronic Kidney Disease Epidemiology Collaboration formula [71] suggests an important relationship between renal ectopic fat and renal function decline. Measurement of perirenal/sinus fat, especially by noninvasive, quick and safe ultrasound or alternatively by more demanding and expensive gold-standard methods such as computed tomography or MRI, might serve to identify obese patients who are at higher risk of obesity-related hypertension, require more aggressive treatment and may benefit the most from bariatric surgery.

### Perivascular fat

Normally, perivascular adipose tissue produces protective nitric oxide (NO) and adiponectin, whereas, in the obese state, perivascular fat secretes proinflammatory mediators that can lead to hypertension [72, 73]. In mice with BMP4 (bone morphogenetic protein 4 belonging to the TGF-beta superfamily) knockout in perivascular fat, high fat diet-induced hypertension is potentiated by reduced NO release and increased reactive oxygen species production, which enhances vasoconstriction and endothelial dysfunction [74]. Furthermore, increased expression of angiotensinogen and AngII suggests that activated perivascular fat can contribute to increase BP in obesity by stimulating the local RAAS. In the model of sympathetic overdrive, norepinephrine stimulated α1A-adrenoceptor (α1A-AR) expression in mesenchymal stromal cells in perivascular fat. This effect correlated with patients’ BP, suggesting that norepinephrine potentiates vasoconstriction via increasing α1A-AR expression, a pathophysiological mechanism possibly contributing to increase systemic BP [75]. Moreover, α1A-ARs were specifically activated by beta3-adrenoceptors, revealing the potential therapeutic role of their antagonism in obesity-related hypertension [75].

## SYMPATHETIC NERVOUS SYSTEM OVER-ACTIVATION

The central nervous system integrates neurohumoral signals mainly in the paraventricular nucleus of the hypothalamus [76–79] and differentially modulates sympathetic output to various tissues based on body fat distribution [5]. Obesity, in particular visceral obesity, is associated with mild activation of the SNS [80, 81] and increased sympathetic nerve activity may in turn increase renal sodium reabsorption and renin release [26], even without evident effects on kidney perfusion (Table 1).

**Table 1:** Evidence for SNS activation in obesity.

Authors, year	Experimental/clinical study	Finding
Rumantir *et al*., 1999 [195]	Lean and obese normotensive and hypertensive subjects	Increased renal, but not cardiac, norepinephrine overproduction in obese vs lean subjects
Vaz *et al*., 1997 [196]	Lean and obese subjects	
Wofford *et al*., 2001 [197]	Lean and obese hypertensive subjects	Higher reduction of BP by pharmacologic inhibition of adrenergic receptors in obese vs lean subjects
Grassi *et al*., 1995 [198]	Lean and obese normotensive subjects	Elevated muscle SNA in obese vs lean subjects
Alvarez *et al*., 2002 [199]	Men with wide range of visceral obesity	
Holwerda *et al*., 2023 [200]	Lean and obese normotensive subjects	
Shibao *et al*., 2007 [201]	Lean and obese subjects with a wide range of BP	Higher reduction of BP by pharmacologic ganglionic blockade in obese vs lean subjects
Muntzel *et al*., 2012 [202]	Female Wistar rats fed a HFD	Increased lumbar or renal SNA in chronic sympathetic nerve recordings of rodents fed a HFD
Armitage *et al*., 2012 [148]	Rabbits fed a HFD	
Lim *et al*., 2016 [203]	Rabbits fed a HFD	
Kassab *et al*., 1995 [204]	Dogs fed a HFD	Bilateral RDN prevented the development of hypertension in animals fed a HFD
Nazari *et al*., 2021 [205]	Male rats fed a HFD	

HFD, high-fed diet; RDN, renal denervation; SNA, sympathetic nerve activity.

Multiple factors contribute to the stimulation of SNS in obesity. Insulin has traditionally been associated with sympathetic overdrive in obesity, but this association has been recently challenged by evidence that muscle sympathetic nerve activity does not appear to correlate with plasma insulin and glucose levels or homeostatic model assessment of insulin resistance (HOMA-IR) [82]. On the other hand, increased leptin activity, impaired chemoreceptor and baroreceptor reflexes, renal ectopic fat and activated RAAS appear to substantially contribute to SNS overactivation in obesity (Fig. 1) [66, 80, 83, 84].

### Leptin

**Figure 3:**
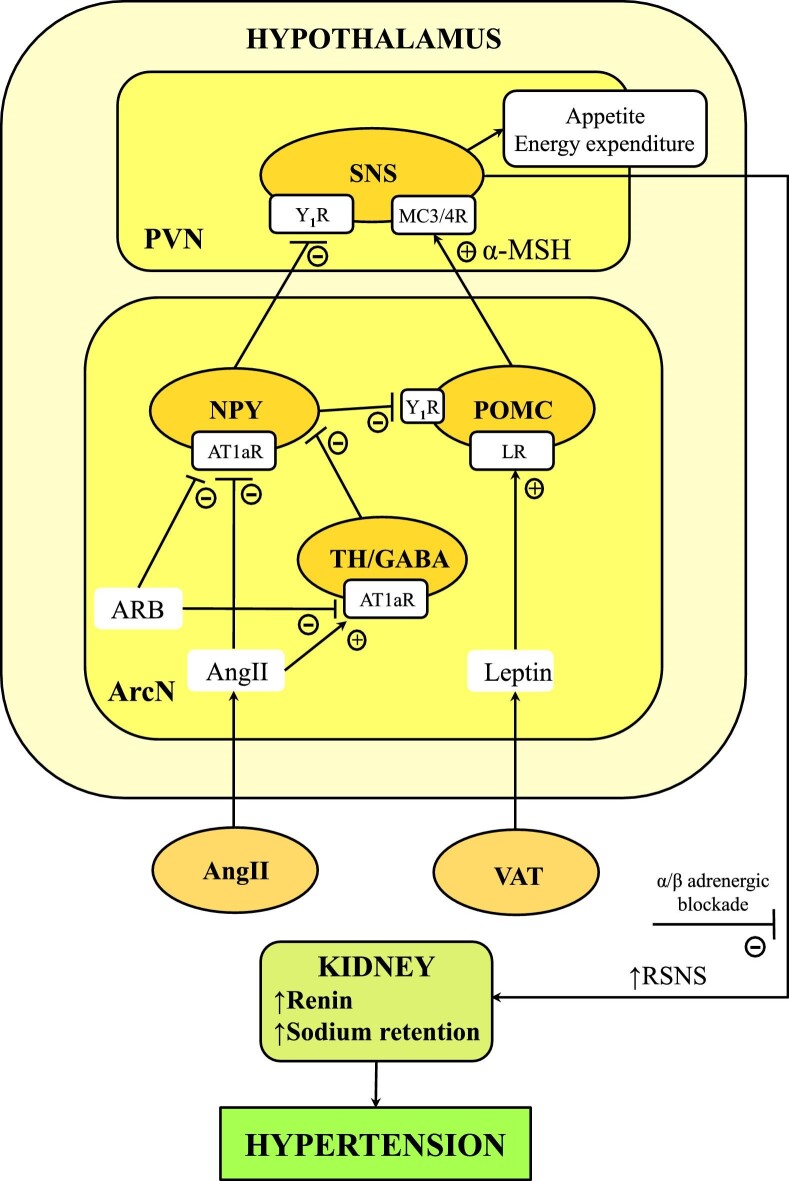
Hypothetical model of leptin sympatho-excitatory action and its synergism with AngII in the arcuate nucleus. LR, leptin receptor; PVN, paraventricular nucleus; RSNS, renal sympathetic nervous system; TH, tyrosine hydroxylase; VAT, visceral adipose tissue; Y_1_R, neuropeptide Y receptor.

The adipocyte-derived hormone leptin, discovered in 1994 by scientists led by Friedman [85], is a major regulator of appetite, energy expenditure and body weight [86–88]. Leptin also stimulates the SNS and increases BP and could be an important link between obesity and hypertension [5, 66]. Leptin stimulates pro-opiomelanocortin (POMC) neurons in the hypothalamic arcuate nucleus (ArcN) that projects to the periventricular nucleus and releases α-melanocortin stimulating hormone (α-MSH), which activates melanocortin receptors (MC3/4R) on presympathetic neurons (POMC-MC3/4R pathway) (Fig. 3) [89–92]. Beyond the regulation of appetite and energy expenditure, the final result is an increase in SNS and BP, an effect that is abolished by α/β-adrenergic blockade [93, 94]. Conversely, MC4R antagonism or genetic disruption of MC4R causes hyperphagia, increased adiposity, insulin resistance and dyslipidemia without affecting BP, a finding that further confirms that insulin resistance and hyperinsulinemia are not the main drivers of obesity-induced hypertension [95–97]. In obesity, the SNS-stimulating action of leptin depends on a functional AngII type 1a receptor (AT1aR) [98–100], which tonically suppresses inhibitory neuropeptide Y (NPY) input to POMC neurons within the ArcN and/or the presympathetic neurons in the periventricular nucleus. AngII inhibits NPY directly and/or indirectly through local interneurons that express tyrosine hydroxylase and gamma-aminobutyric acid (GABA) [101]. Thus, AngII-AT1aR results in a ‘gatekeeper’ of leptin-induced sympatho-excitation [99]. Consistently, sympathetic-excitatory and pressor responses to ArcN leptin nanoinjections in rats were eliminated by local AT1aR blockade with angiotensin receptor blockers (ARBs) [99], suggesting that in obesity the antihypertensive effect of ARBs can be amplified by inhibition of the sympatho-excitatory effect of leptin.

Obese patients develop ‘selective’ leptin resistance that is associated with attenuated anorexic and metabolic effects without appreciable effects on SNS and BP [102–104]. Therefore, leptin antagonism therapy is not expected to help in reducing BP, whereas it might even worsen obesity. However, in obese mice, the administration of leptin-neutralizing antibodies was found to restore hypothalamic sensitivity to leptin, reduce food intake, increase energy expenditure and improve insulin sensitivity [105]. Thus, there could be a leptin concentration threshold and its surmounting could further increase desensitization of hypothalamic neurons contributing to weight gain. Conceivably, partial leptin inhibition could restore leptin sensitivity and reduce obesity.

Elevated leptin levels can also induce mitochondrial dysfunction in adipocytes, which in turn may result in further leptin overproduction [106, 107]. This self-reinforcing cycle can stimulate SNS and increase BP and could be a further link between leptin and obesity-related hypertension. Indeed, mitochondrial dysfunction in adipocytes appears to contribute to hypertension in obese patients [107, 108]. This hypothesis is corroborated by evidence that in mice, simultaneous double calcium channel TRPV1 and uncoupling protein 1 (UCP1) knockout induces severe obesity and hypertension [109]. Hypertension results from impaired mitochondrial calcium uptake and subsequent production of reactive oxygen species. Thus, regulation of mitochondrial calcium homeostasis might represent a new therapeutic target for obesity-related hypertension.

Elevated leptin causes sodium retention also through direct action on the renal tubules and stimulation of aldosterone (Fig. 1) [110]. Obesity is also characterized by increased activity of neprilysin released from adipocytes [111, 112] and kidneys under sympathetic nervous activation, leading to a decrease in natriuretic peptides, which amplifies aldosterone and leptin-dependent renal-pressure natriuresis impairment. Thus, the interplay of the leptin, aldosterone and neprilysin axis stimulates both the SNS and the RAAS, as observed in obese individuals with heart failure [113].

### Paraventricular nucleus sympatho-excitation

In addition to leptin, multiple factors might be involved in paraventricular nucleus signalling to mediate SNS activation and BP increase in obesity. Increased AngII via AT1R can stimulate NADPH oxidase, which increases the production of reactive oxygen species and activates the SNS [78, 114]. Toll-like receptor 4 and extracellular signal-regulated kinase (ERK) activation in the paraventricular nucleus are major factors in the lipopolysaccharide (LPS)-induced increase in sympathetic nervous activity [115]. Therefore, inhibiting the effect of these factors may be a good strategy to attenuate SNS activity and obesity-related hypertension. Table 2 shows evidence for a modulatory role of intermedin and adrenomedullin (cardiovascular peptides from the CGRP superfamily [116–121]) in BP reduction through attenuation of sympatho-excitation in obesity.

**Table 2:** Recent evidence for a modulatory role of intermedin and adrenomedullin on sympatho-excitation and BP reduction.

Authors, year	Experimental study	Finding
Kang *et al*., 2019 [206]	Rats with obesity-HT induced by a HFD for 12 weeks	IMD, injected into the PVN, attenuates SNA and hypertension via AMD receptors in PVN, and reduces AngII-induced increase in SNA by inhibiting NADPH oxidase activity and ERK activation
Sun *et al*., 2019 [115]	Rats with obesity-HT induced by a HFD for 12 weeks	IMD, injected into the PVN, reduces SNA and BP via activation of ADM, which attenuates ERK-LPS-TLR4 sympatho-excitation
Wang *et al*., 2023 [207]	Rats with obesity-HT induced by a HFD for 12 or 16 weeks	ADM, injected into the PVN, attenuates adipose afferent reflex and sympathetic overactivation, and decreases BP via NO-cGMP-GABA pathway

AMD, adrenomedullin; HFD, high-fat diet; IMD, intermedin; LPS, lipopolysaccharide; obesity-HT, obesity-related hypertension; PVN, paraventricular nucleus; SNA, sympathetic nerve activity; TLR4, Toll-like receptor 4.

### Carotid bodies and chemoreflexes

Peripheral chemoreceptors such as the carotid bodies can be implicated in hypertension development in relation to increased sympathetic nervous activity [122–127]. Indeed, carotid bodies denervation inhibits BP increase induced by leptin infusion in lean and obese mice whereas the expression of leptin receptor in the carotid bodies leads to hypertension in leptin receptor–deficient db/db mice [128, 129]. In the glomus cells of carotid bodies, leptin acts through the long isoform of leptin receptor, the only leptin receptor presenting intracellular signalling, which increases the expression of transient receptor potential cation channel subfamily M member 7 (Trpm7) and transmits chemosensory input from carotid bodies to medullary centres and increases BP [128]. Inhibition of *Trpm7* gene expression in carotid bodies significantly reduced BP [128]. Furthermore, a single injection of FTY720 hydrogel, a novel extended-release TRPM7 blocker, into mice carotid bodies lowered BP for at least 3 weeks [129]. Thus, leptin induces hypertension by acting also in the carotid bodies, which opens the perspective for TRPM7 blockers as a potential therapy in obesity-related hypertension.

Glucagon-like peptide 1 receptors (GLP-1Rs) are also expressed in the carotid bodies of spontaneously hypertensive rats. Their depletion increased chemoreflex-evoked sympathetic drive, whereas GLP-1R agonists (GLP-1RAs) acutely suppressed the chemoreflex-evoked sympathetic and BP responses [130]. This reasonably explains the antihypertensive effect of GLP-1RAs observed in spontaneously hypertensive rats [131] and T2DM patients [132–134].

Hypoxemia may also result in carotid bodies activation and consequent BP increase (Fig. 1). Obesity increases metabolic rate, impairs respiratory mechanics and may cause chronic hypoxemia [135–138] or obstructive sleep apnoea [139–141]. In hypoxemic/eucapnic obese dogs, chronic hypoxemia has been shown to stimulate chemoreceptors in the carotid bodies, increasing sympathetic nervous activity and BP [142]. In patients with CVD and obstructive sleep apnoea, treatment with continuous positive airway pressure (CPAP) suppresses chemoreflex activity and reduces sympathetic nervous activity, heart rate and BP [141, 143, 144]. The combination of CPAP and weight loss synergistically lowers BP in obese patients with obstructive sleep apnoea [145]. However, to which extent oxygen therapy could affect the multiple pathophysiologic mechanisms that underlie obesity-related hypertension is still unclear. Carotid bodies resection can also reduce muscle sympathetic nerve activity in humans. Notably, bilateral resection could help reducing BP, but could also worsen hypoxemia. Its potential therapeutic role to alleviate abnormal sympathetic nervous activity in obesity-related hypertension requires further investigation [146, 147].

### Baroreflex sensitivity

Baroreflex sensitivity is a homeostatic mechanism that maintains stable BP by continuous signals to the brain through mechanosensitive endings embedded in the aortic arch or carotid sinus. Baroreflex sensitivity regulates BP by controlling peripheral vascular tone and cardiac output via the parasympathetic and sympathetic autonomic nerves, and their dysregulation is critical for the development of resistant hypertension. Obesity [148–150], especially visceral obesity [151], is associated with reduced baroreflex sensitivity (Fig. 1). Conversely, electrical baroreceptor activation inhibits sympathetic nervous activity without significant long-term counteractive renin secretion, improves sympatho-vagal balance, reduces arterial resistance and stiffness, increases renal natriuresis and lowers BP [149, 152–157]. Notably, baroreceptor activation lowers BP even in the absence of increased baseline sympathetic nervous activity and after renal denervation. Baroreceptor activation also reduces glomerular hypertension and hyperfiltration in obesity, which may be protective against progressive renal function decline [153]. The idea of treating hypertension by modulating baroreceptor activation rose in the 1960s, but the procedure was impractical due to major technical limitations, and was only used for research purposes [158–160]. In more recent years a device-based approach (the Rheos system) consisting of an implanted pulse generator in the thoracic region that delivers electrical pulses to the carotid baroreceptors has been developed and used in humans with resistant hypertension, but with poor efficacy and major safety issues related to the implantation procedure [155]. Better results with more effective BP reduction have been subsequently obtained with a second-generation less invasive and safer Barostim neo™ system [156, 157]. However, randomized controlled trials will be needed to definitively test the role of baroreceptors in the pathogenesis and treatment of obesity-related hypertension refractory to pharmacological therapy.

### Renal sympathetic nervous system

Renal SNS consists of efferent (sympathetic) and afferent (sensory) fibres that coordinate renal function, central haemodynamics and arterial BP. Sympathetic nerves increase renin secretion, promote sodium reabsorption and regulate renal blood flow [161]. Sensory nerves densely innervate the pelvic wall, renal vasculature and tubules. They respond to both mechano- and chemo-sensitive stimuli, such as altered renal perfusion pressure and circulating chemokines, and project signals to the central nervous system [162]. In obesity, the activated renal SNS mediates much of the BP-elevating mechanisms related to sympathetic nerve activation (Fig. 1) [163, 164]. The pathogenic role of renal SNS overactivation in obesity-related hypertension is confirmed by experimental and clinical studies showing that renal denervation lowers BP and attenuates sodium retention [165, 166]. Consistently, results of multiple sham-controlled, catheter-based renal denervation trials showed the BP-lowering efficacy (over 24 h) of radiofrequency and ultrasound renal denervation in hypertensive patients, including patients with resistant and/or obesity-related hypertension, independent of concomitant antihypertensive pharmacological treatment [167–172]. However, a very recent meta-analysis [173], evaluating muscle SNS responses to renal denervation in patients with resistant hypertension, showed that denervation was significantly associated with central sympathoinhibition independently of BP reduction. Indeed, finding that renal denervation in untreated hypertensive patients was associated with decreased plasma renin activity and aldosterone levels at 3 months compared with a blinded sham-controlled group [174], can be taken to suggest that other factors could be associated with the BP-lowering effect of renal denervation. In this context, plasma renin activity could play a role as suggested by evidence that higher baseline renin activity was associated with greater denervation-induced BP reduction [175].

## RENIN–ANGIOTENSIN–ALDOSTERONE SYSTEM ACTIVATION

The circulating RAAS was discovered in 1898 by Tigerstedt and Bergman, who reported the pressor effect of a renal extract named renin. In the 1930s Harry Goldblatt and co-workers significantly contributed to the study of the RAAS demonstrating its relevance in experimental hypertension. Since 1936, two separate research groups independently proposed that renin acts enzymatically on a plasma protein, leading to the production of another pressor agent, eventually called ‘angiotensin’ in 1958 [176]. Thus, systemic RAAS was identified as a key regulator of salt and water balance and BP [177]. Then, many local RAASs were subsequently identified in heart, kidney, adipose tissues, brain, immune system and vasculature [178]. The RAAS consists of two pathways: a pro-inflammatory pathway involving the angiotensin-converting enzyme (ACE)/AngII/AT1/Aldosterone/mineralcorticoid receptor (MR) axis and an anti-inflammatory pathway involving the AT2/ACE2/Ang1–7/Mas receptor axis [177, 178]. The prevalence of pro-inflammatory pathway appears to contribute to hypertension and excess cardio-renal risk in obesity.

Obesity is associated with a mild-to-moderate increase in both systemic and local adipose RAAS activity despite sodium retention and elevated BP, factors that should physiologically inhibit renin secretion, AngII formation and aldosterone secretion [66, 179]. Although adipocyte-specific angiotensinogen production has been reported to be a major source of circulating AngII [180], the contribution of adipose AngII to obesity-related hypertension is still unclear [66]. Elevated AngII levels in obesity may be driven by sympathetic nervous activation, kidney compression and perhaps increased production of adipokines [181, 182]. In obesity, AngII sensitivity is elevated [183], and even a minimal increase in AngII stimulates sodium reabsorption, efferent arteriolar constriction and glomerular pressure elevation leading to glomerular hyperfiltration, a haemodynamic change that heralds kidney damage [184] (Fig. 2). Moreover, obesity is a risk factor for CKD, even independently of its effects on BP [67, 185]. In turn, CKD progression further exacerbates obesity-related hypertension and favours resistance to BP-lowering treatments [186].

**Figure 4:**
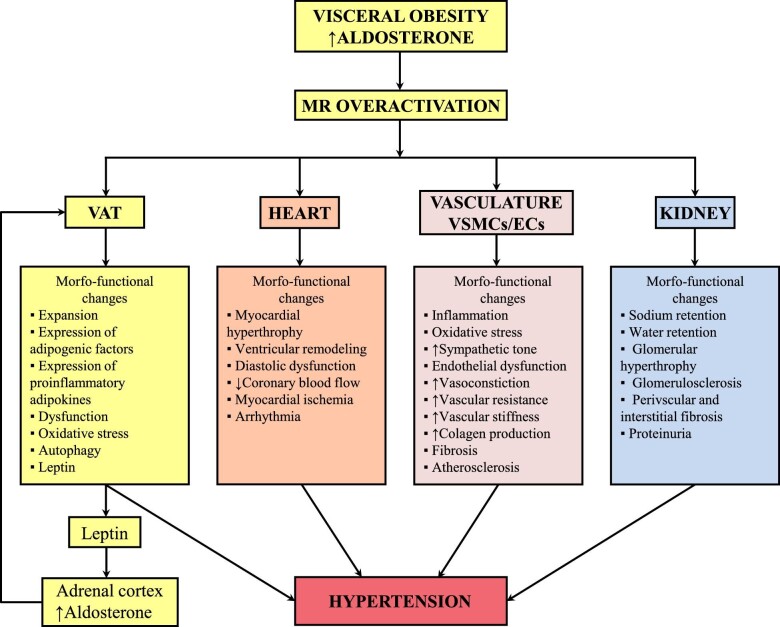
Hypothetical model of the morpho-functional changes in cardiorenal, vascular and visceral adipose tissue under MR overactivation and their association with hypertension. ECs, endothelial cells; VAT, visceral adipose tissue; VSMCs, vascular smooth muscle cells.

Aldosterone levels are positively associated with visceral fat, independent of plasma renin activity [187]. The interaction between aldosterone and visceral fat is bidirectional and is promoted by leptin. Adipocyte-derived leptin stimulates aldosterone secretion from the adrenal cortex (Figs 1 and 4) [188]. Accordingly, the finding that in obese leptin-deficient (ob/ob) and leptin receptor–deficient (db/db) mice plasma aldosterone levels are in normal range confirms the crucial role of leptin in sustaining increased aldosterone production in obesity [188]. In turn, aldosterone enhances MR-induced expression of leptin and other cytokines [189], modulating inflammatory status and visceral fat dysfunction and contributing to the development of obesity-related hypertension. Interestingly, the effect of leptin on aldosterone secretion and BP is sex-dependent as demonstrated by evidence that obesity induced by neuronal leptin-receptor deletion is associated with increased plasma aldosterone and BP in female mice, whereas in male mice aldosterone and BP levels do not increase despite severe obesity [86]. MR blockade reduces BP only in obese hyperleptinemic female mice, whereas in obese male mice, BP is reduced only by adrenergic receptor blockade [190, 191]. Finally, in female mice, leptin induces endothelial dysfunction and hypertension via the leptin–aldosterone–endothelial MR axis (Fig. 1) [192].

In obesity, MR overactivation can be observed even in the absence of high plasma aldosterone (Table 3) [67, 193]. The role of MR and RAAS blockade in the management of obesity-related hypertension will be discussed in Part 2.

**Table 3:** Possible mechanisms for aldosterone-independent MR activation and its role in the development of hypertension and renal injury.

Authors/year	Experimental/clinical study	Finding
Massaad *et al*., 1999 [208]	*In vitro* transient transfection assays in HepG2 cells	Protein kinase A modulates transcriptional activity of the human MR
Nagase *et al*., 2007 [209]	SHR/cps fed a high-sodium diet for 4 weeks	High-salt loading reduces circulating aldosterone but causes activation of renal MR at least in part by induction of oxidative stress, exacerbates proteinuria and renal injury. Eplerenone effectively improves nephropathy
Shibata *et al*., 2008 [210]	*In vitro* transfection assays in HEK 293 cells	Rac1 activation induces nuclear translocation of MR, leading to the increased transcriptional activity of MR-dependent genes
	Animals: Arhgdia^–/–^ mice	Increased Rac1 and MR signalling in the kidney is associated with heavy albuminuria and podocyte damage
		MRA suppresses albuminuria and histological changes
Shibata *et al*., 2011 [211]	Salt-sensitive and salt-insensitive (Dahl-S/Dahl-R) rats	High-salt loading activates Rac1 in the kidneys of Dahl-S rats stimulating MR that leads to BP elevation
		Rac1 inhibition prevents hypertension and renal damage
Tapia-Castillo *et al*., 2015 [212]	Adult Chilean subjects with high and low sodium intake	In subjects on high sodium intake, increased Rac1 expression is associated with increased expression of MR, NGAL (kidney damage), NF-κB (inflammation), and HO-1 (ROS formation)
Hall *et al*., 2015 [5]Hall *et al*., 2021 [67]	Obese dogs (referenced)	Decreased 11b-HSD2 (co-localized with the MR in renal collecting tubules that normally converts active cortisol to inactive cortisone) might permit cortisol to activate MR in obesity
Jo *et al*., 2023 [213]	*In vitro* coimmunoprecipitation assays in HEK293T cellsAnimals: diabetic db/db mice	O-GlcNAcylation of the MR directly increases protein levels and transcriptional activities of the receptor under high-glucose conditions *in vitro* and *in vivo*

11b-HSD2, 11b-hydroxysteroid dehydrogenase type; HO-1, hemoxigenase-1; MRA, mineralocorticoid receptor antagonist; NF-κB, nuclear factor-κB; NGAL, neutrophil gelatinaseassociated lipocalin; O-GlcNAcylation, O-linked-N-acetylglucosamine modification; rRac1, a member of the Rho family GTPases; SHR/cps, rat model of metabolic syndrome.

Furthermore, soluble (pro)renin receptor (sPRR), a cleavage product of full-length PRR, has been suggested to be involved in intrarenal RAAS activation, ENaC expression, endothelial dysfunction and impaired baroreflex response, and might be therefore a potential target of novel pharmacological interventions for obesity-related hypertension [194].

## CONCLUSIONS

Obesity, especially when associated with increased visceral and ectopic fat expansion, is a major cause of hypertension and related cardiovascular and kidney injury. Obesity-related hypertension is initiated by increased renal sodium reabsorption and plasma volume expansion due to renal compression by perirenal/sinus fat and moderate increases in systemic/renal SNS and RAAS activity sustained by a complex interplay among hyperleptinemia, AngII, intermedin, adrenomedullin, and impaired baroreceptor and chemoreceptor reflexes. This manuscript overviews a series of pathophysiological mechanisms involved in the pathogenesis of obesity-related hypertension—such as leptin resistance, impaired baroreceptor and chemoreceptor reflexes, increased renal sympathetic nervous activity, mitochondrial dysfunction, the regulatory role of intermedin, adrenomedullin and sPRR—that could be the target of specific and selective therapeutic interventions in the innovative context of precision medicine. Therapeutic options for obesity-related hypertension will be discussed in Part 2 of the review.

## Data Availability

No new data were generated or analysed to support this review.
